# A Wearable Proprioceptive Stabilizer (Equistasi®) for Rehabilitation of Postural Instability in Parkinson’s Disease: A Phase II Randomized Double-Blind, Double-Dummy, Controlled Study

**DOI:** 10.1371/journal.pone.0112065

**Published:** 2014-11-17

**Authors:** Daniele Volpe, Maria Giulia Giantin, Alfonso Fasano

**Affiliations:** 1 Department of Physical Medicine & Rehabilitation, S. Raffaele Arcangelo Fatebenefratelli Hospital, Venice, Italy; 2 Morton and Gloria Shulman Movement Disorders Clinic and the Edmond J. Safra Program in Parkinson’s Disease, Toronto Western Hospital and Division of Neurology, University of Toronto, Toronto, Ontario, Canada; University of Glasgow, United Kingdom

## Abstract

**Background:**

Muscle spindles endings are extremely sensitive to externally applied vibrations, and under such circumstances they convey proprioceptive inflows to the central nervous system that modulate the spinal reflexes excitability or the muscle responses elicited by postural perturbations. The aim of this pilot study is to test the feasibility and effectiveness of a balance training program in association with a wearable proprioceptive stabilizer (Equistasi) that emits focal mechanical vibrations in patients with PD.

**Methods:**

Forty patients with PD were randomly divided in two groups wearing an active or inactive device. All the patients received a 2-month intensive program of balance training. Assessments were performed at baseline, after the rehabilitation period (T1), and two more months after (T2). Posturographic measures were used as primary endpoint; secondary measures of outcome included the number of falls and several clinical scales for balance and quality of life.

**Results:**

Both groups improved at the end of the rehabilitation period and we did not find significant between-group differences in any of the principal posturographic measures with the exception of higher sway area and limit of stability on the instrumental functional reach test during visual deprivation at T1 in the Equistasi group. As for the secondary outcome, we found an overall better outcome in patients enrolled in the Equistasi group: 1) significant improvement at T1 on Berg Balance Scale (+45.0%, p = .026), Activities-specific Balance Confidence (+83.7, p = .004), Falls Efficacy Scale (−33.3%, p = .026) and PDQ-39 (−48.8%, p = .004); 2) sustained improvement at T2 in terms of UPDRS-III, Berg Balance Scales, Time Up and Go and PDQ-39; 3) significant and sustained reduction of the falls rate.

**Conclusions:**

This pilot trial shows that a physiotherapy program for training balance in association with focal mechanical vibration exerted by a wearable proprioceptive stabilizer might be superior than rehabilitation alone in improving patients’ balance.

**Trial Registration:**

EudraCT 2013-003020-36 and ClinicalTrials.gov (number not assigned)

## Introduction

Parkinson’s disease (PD) is a progressive neurological condition associated with reduced physical activity and poor mobility. Postural instability severely affects the conditions of these patients because it is associated with an increased risk of falls, immobility, hospitalization and the need for long-term care [1], [2], overall reducing the health-related quality of life [3].

The pathophysiology of postural instability in PD is not fully understood as it probably depends from a complex interaction between compensatory strategies and the impairment caused by the disease at different levels of the nervous system [4], [5]. Several posturographic studies investigating the centre of pressure (COP), in both static and dynamic conditions, have showed that PD patients sway significantly more than healthy subjects because they tend to exceed their limits of stability to a much greater extent [6]. On the other hand, early [7] and recent [8] studies have demonstrated that PD patients have a reduced limit of stability particularly during dynamic conditions, thus supporting the hypothesis that an important role is played by an impairment in appropriately scaling the postural reactions in response to perturbations [9].

In a gravity environment, with a firm base of support, healthy subjects mainly rely on somatosensory information in order to maintain an upright posture [10]. Accordingly, artificially impairing proprioception worsens postural stability and particularly reduces the COP displacements in response to external perturbations during visual deprivation [11]. Muscle spindles endings are extremely sensitive to externally applied vibrations, and under such circumstances they convey proprioceptive inflows to the central nervous system that modulate the spinal reflexes excitability [12], [13], [14] as well as posture [15], [16], [17] or the muscle responses elicited by postural perturbations [9], [18], [19]. Similar protocols have been applied in PD patients during either static [20] or dynamic [21] conditions, obtaining responses similar to healthy subjects, in keeping with a normal integration of the proprioceptive inflow. Notwithstanding, it is known that the postural control of PD patients mainly relies on visual cues, possibly compensating for a proprioceptive impairment [22]. In keeping with a role for proprioceptive impairment in PD, Valkovic et al. [23], [24] documented a defective scaling and habituation of postural reactions during either neck or legs vibration, the extent of these abnormalities being correlated with disease progression.

Although there is growing evidence showing that physical activity and exercise programs can improve strength [25], balance [26], [27], mobility [28] and quality of life [3], [29] in patients with PD, most studies have shown limited long-term benefits despite short-term gains [30], [31], [32]. Therefore, there is a cogent need to find effective and innovative methods for training balance in people with this debilitating and progressive disease [33], [34].

Alternate vibratory stimulation on trunk muscles has been used for therapeutic purposes in PD, providing an improvement of trunk sway [35] or gait [21]. In particular, Nanhoe-Mahabier et al. [35] have recently investigated the effect of balance training combined with artificial vibrotactile biofeedback on the trunk sway of PD patients and have found that feedback group had a significantly greater reduction in roll and pitch sway angular velocity, thus resulting in a beneficial effects on trunk stability; authors concluded that further studies should examine if these effects increase further after more intensive training and how long these persist after training has stopped [35]. Therefore, we can argue that combining a perturbation-based training in association with a wearable postural stabilizer (WPS) providing prolonged muscle mechanical vibrations could improve postural stability in PD.

**Figure 1:**
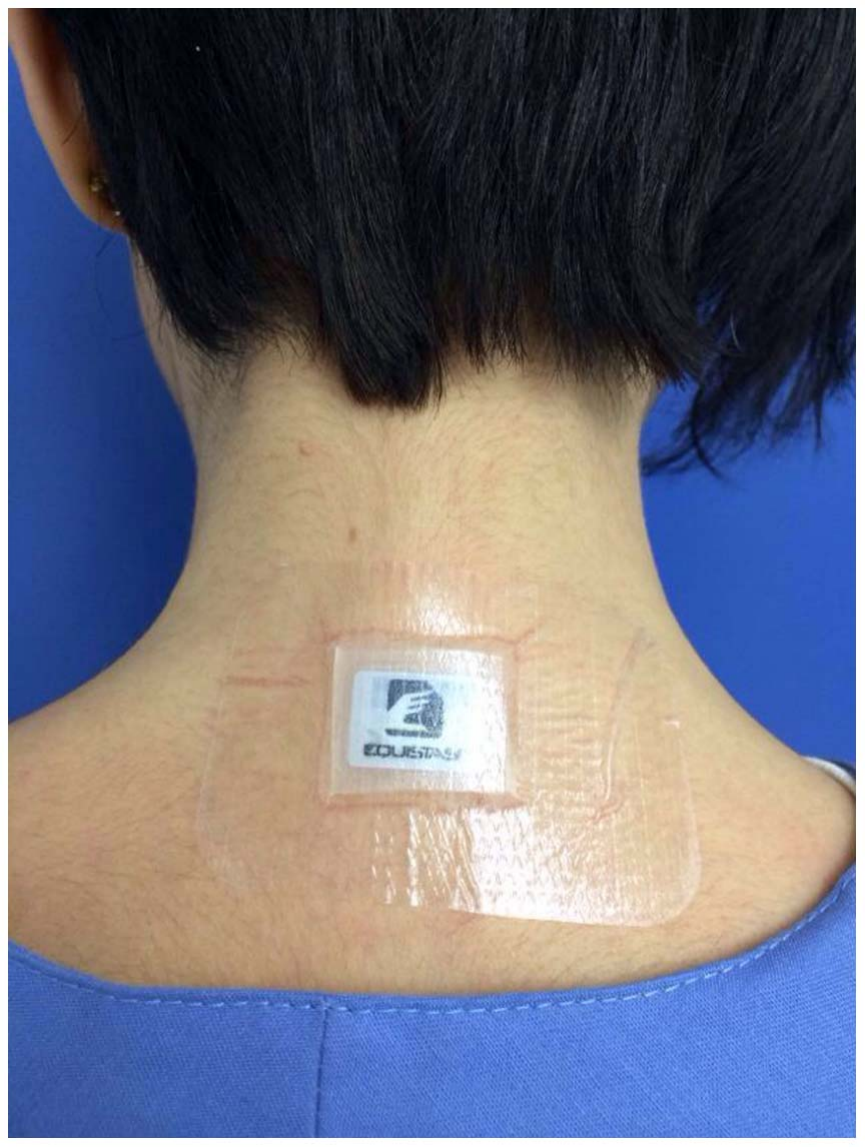
The wearable postural stabilizer (Equistasi) employed in the present study.

The present phase II double-blind, double-dummy randomized controlled trial (RCT) tests the feasibility, safety and effectiveness of a standard balance training program combined with the use of a WPS (Equistasi, Milan, Italy). Equistasi is a registered (class 1, ministerial code n. 342577 on 05/08/2010) medical device consisting in a rectangular plate measuring 10×20×0.5 mm and with a weight of 0.17 gr (Figure 1). The device is exclusively composed by nanotechnology fibers that transform the body temperature into mechanical vibratory energy (<0.8N, 9000 Hz) able to generate a variation of muscle length of max 0.02 mm [36], by far within the safety limit (0.12 mm) found to be harmful for human muscles [37].

The present RCT will enable us to examine whether enhancing balance training using a WPS in a rehabilitation setting leads to a clinically meaningful effect in PD patients with balance impairment and is safe, i.e. it does not worsen postural stability.

## Methods

### Design

**Figure 2:**
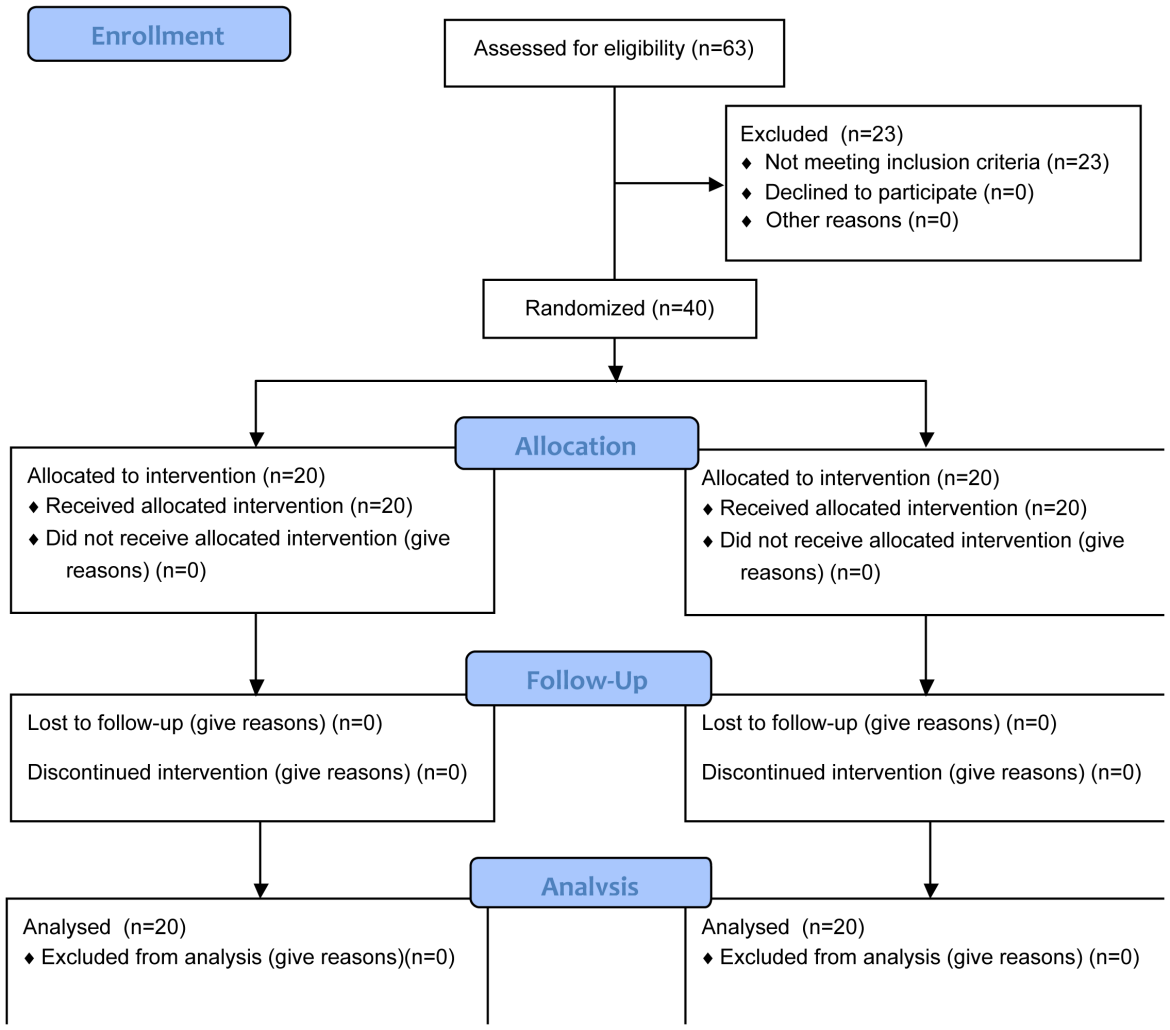
The CONSORT flow diagram for this study.

We conducted a double-blind, double-dummy, parallel group RCT with a focus on clinical measures of balance as primary outcome. The study protocol and supporting CONSORT checklist are available as supporting information (see Protocol S1 and Checklist S1). After screening and enrolment, forty patients were monitored for 2 months in order to record the falls rate. Afterward, participants were randomized to receive a 2-month intensive (see below) program of balance training while wearing a WPS (Equistasi) or the identical training program while wearing a placebo device identical to the active one (Figure 2). Patients were recruited from the Neurorehabilitation Unit of “S. Raffaele Arcangelo” Hospital in Venice, Italy. The trial was approved by the hospital ethics committee (C.E.O.C. Brescia Italy, ref 35/2013) and was registered online at EudraCT (n. 2013-003020-36) and at ClinicalTrials.gov (number not assigned and delayed in being posted online due to the FDA restrictions for devices unapproved in U.S.). Written informed consent was obtained from the participants or from their spouses if they scored less than 25/30 on the Mini Mental State Examination (MMSE) [38].

### Participants

Participants were eligible for inclusion if they consented to participation, had PD diagnosed according to the current criteria [39], Hoehn and Yahr [40] stage ≥2 on levodopa, and history of at least one fall in the past. Exclusion criteria were: medication-induced dyskinesias (to avoid confounding effects on force platform assessments), presence of co-morbidities preventing mobility or safe exercise (including clinically evident neuropathy and major medical conditions such as malignancies), history of deep brain stimulation (DBS) surgery or other conditions affecting stability (e.g. poor visual acuity or vestibular dysfunction), Hoehn and Yahr stage ≥4 on levodopa, and inability to travel to the physiotherapy venues.

### Randomization and blindness

A blocked stratified randomization procedure conducted by a third party and based on the Hoehn & Yahr score was used to allocate participants to one of the two treatment groups (i.e. physiotherapy with or without an active WPS). The two trained assessors and patients were blinded to the group allocation during the whole duration of the study. The study coordinator responsible for WPS placing (M.G.G.) was not blinded to group allocation, but she was not involved in rehabilitation procedures or outcome assessment. The therapists providing the interventions were blinded and not involved in other aspects of the trial (i.e., aims, hypotheses or predictions of the study were not disclosed). Both active and placebo WPSs were identical and did not cause any recognizable sensory sensation, thus guarantying patients’ blindness. To test the quality of blinding procedures, the trained assessors were asked to guess the group allocation at the end of the trial and they only guessed 40% of the group assignments.

### Intervention

Participants of the two groups received the same 60-minute physiotherapy daily session 5 days a week for 2 months at the S. Raffaele Hospital of Venice, Italy. Table S1 details the type of daily physiotherapy provided by the hospital physiotherapists. The physiotherapy protocol included 40 minutes’ individual sessions designed to improve balance with a perturbation-based balance training program, where patients were asked to voluntary reach their limit of stability in protected conditions and taught how to correctly activate the postural responses to external perturbations. Exercises were preceded by warming up and stretching exercises and followed by cooling down, each epoch lasting 10 minutes. At the beginning of each session, participants were required to sign a form in order to attest their attendance.

While receiving the same physiotherapy, participants were allocated to two groups:

“**Equistasi**”: each patient wore 3 Equistasi devices (Figure 1), applied over the 7^th^ cervical vertebra and on each soleus muscle tendons. These sites were chosen on the basis of previous studies showing changes of the centre of pressure (COP) induced by either leg or paraspinal muscle vibration [16], [20], [41].
“**Placebo**”: each patient wore 3 inactive devices, applied on the same body sites chosen for the active group.

During the first three weeks of rehabilitation, both groups wore the devices six days a week, 60 (1^st^ week), 120 (2^nd^ week) and 180 (3^rd^ week) minutes a day; during the fourth week onward, they wore the devices for 5 days a week, four hours a day. Devices application was held in the morning, prior to the physiotherapy program; devices were removed on the same day after the necessary time was elapsed, meaning that patients waited in the hospital area after the training session. Medications were unchanged during the whole trial period.

### Outcome Measures

We assessed outcomes at three time points. Baseline (T0) measures were taken within 1 week prior to enrolling. The second assessment (T1) occurred within 1 week after the two-month therapy period. The last assessment (T2) was undertaken two months after T1. T1 and T2 were chosen in order to evaluate the attainment and retention of skills learned during physiotherapy classes [42].

#### Instrumental assessment

As primary measures of outcome for balance, static posturography (stabilometry) was assessed in keeping with current guidelines [43]. The COP sway in the antero-posterior (AP) and medio-lateral (ML) directions was recorded on a force platform (Milletrix model 2.0– Rome, Italy) with an acquisition frequency of 50 Hz. Acquisition was performed during upright stance with the patient barefoot with the feet splayed out at 30 degrees, while keeping the arms alongside the body and staring at a fixed point marked on the wall at a distance of one meter at the height of the glabella of each individual. Data acquisition was performed for 51.2 seconds under each condition [with eyes open (EO) or closed (EC)]. The following parameters were taken into account: mean COP velocity (m/s) and sway area (mm^2^) and path (mm).

The same equipment was used to evaluate an instrumental version of the functional reach test (FRT) [44], by asking the subject to bend forward while maintaining feet planted in a standing position during both EO and EC conditions. The sway area (mm^2^) and the displacement along the AP axis were taken into account; displacements along the ML axis were also recorded but not taken into account for the analysis, given the notion that PD patients display instability principally along the AP one [45] and considering that patients were asked to bend forward along the AP axis.

#### Clinical assessment

Motor impairment was assessed with the parts II (activities of daily living) and III (motor examination) of the Unified PD Rating Scale [46], the Timed Up and Go (TUG) [47], and the Berg Balance Scale (BBS) [48]. Falls Efficacy Scale (FES) [49] and the Activities-specific Balance Confidence (ABC) [50] were administered to measure the fear of falling. Falls were recorded by means of fall diaries of the previous two months. We also quantified health-related quality of life in all participants using the PDQ-39 [51]. Other data collected at baseline included age, gender, body mass index (BMI), disease duration, anti-PD medications expressed as levodopa-equivalent daily dose [52], cognitive status assessed with the MMSE. All adverse events such as injuries, distress and hospital admissions were verified by phone interview and recorded during the trial period.

### Statistical Analysis

This clinical trial used a sample of convenience, with the assumption that 40 participants would be ample to explore safety and feasibility. Given the small sample and the lack of normal distribution of most of the variables on Shapiro-Wilk test, non-parametric statistics were used. Absolute values and magnitude of change were compared in both groups at the three time points by means of Mann-Whitney U test. Treatment effect across time points in each group were explored by means of the Friedman analysis of variance by ranks, and in case of statistical significance post-hoc comparisons were carried out with the Wilcoxon signed-rank test. Categorical variables were compared by means of chi-square test. All values were expressed as median (25^th^ and 75^th^ percentiles), with the exception of figures, where mean and standard deviation were chosen to improve clarity of data presentation. IBM SPSS Statistics ver. 20.0 was used for all statistical analyses. All tests were two-sided with a level of significance set at P<0.05.

## Results

**Figure 3:**
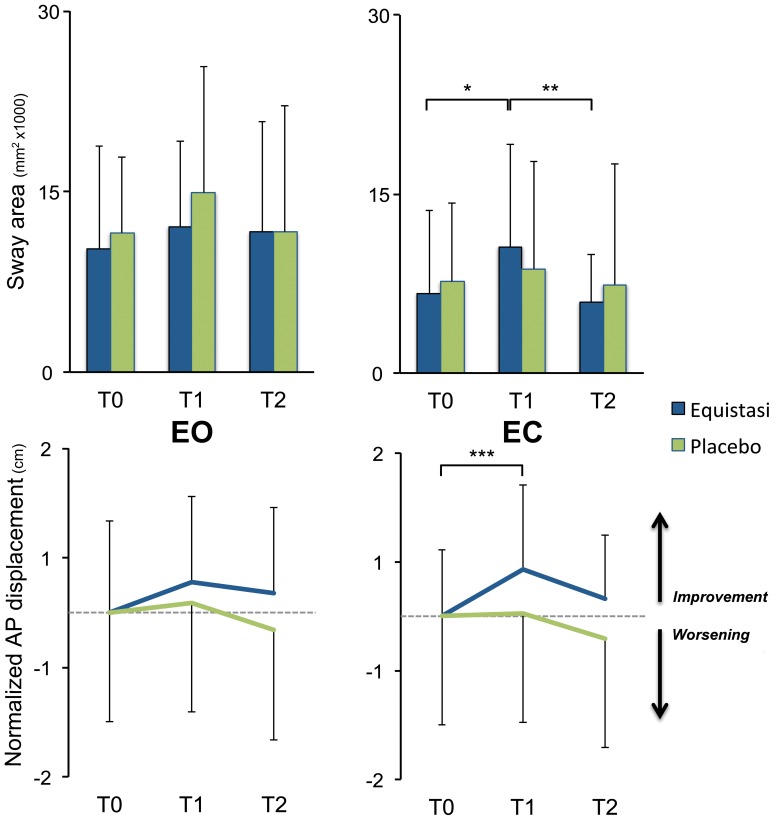
The sway area and the displacement along the AP axis (expressed as value normalized to the baseline) at the instrumental FRT during EO (left panels) and EC (right panels) conditions. Data are presented as mean ± standard deviation. Abbreviations: AP: antero-posterior; EC: eyes closed; EO: eyes open; *: p = .006; **: p = .02; ***: p = .01 (Wilcoxon signed-rank test).

**Figure 4:**
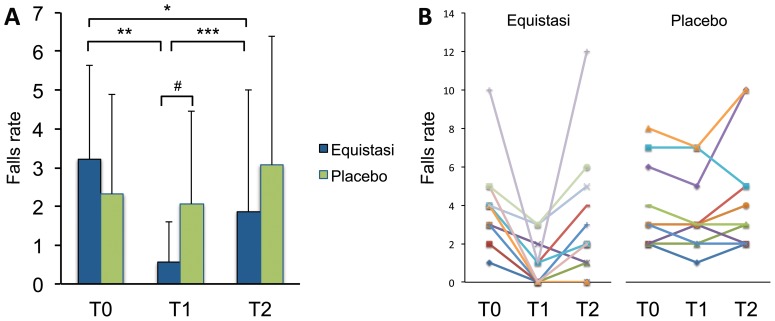
The Falls rate over the 2-month observation period. Data are presented as mean ± standard deviation (A) and individual trends excluding patients with no baseline history of falls (B). Abbreviations: *: p = .03; **: p = .0001; ***: p = .003 (Wilcoxon signed-rank test); #: p = .03 (Mann-Whitney U test).

Twenty subjects were enrolled in each study arm and they did not differ in any of the demographic and baseline clinical and instrumental data (Tables 1–2 and Figures 3–4). For the sample as a whole, 95.6% of intervention sessions were delivered without differences across study arms (2160 and 2220 minutes on average for Equistasi and placebo group, respectively); sessions were not delivered due to personal reasons or due to illnesses not related to PD. No major adverse event or death was observed during the study period.

**Table 1:** Baseline demographic and clinical variables of the two groups enrolled in the study.

	Active group	Placebo group	P
	(n = 20)	(n = 20)	value*
Gender	7M/13F	9M/11F	.747
Age	66.5 (64.0; 78.0)	69.5 (65.0; 73.8)	.947
BMI	24.6 (22.9; 27.0)	27.7 (24.4; 29.0)	.060
H&Y	3.0 (3.0; 3.0)	3.0 (2.0; 3.0)	.429
Disease duration	6.0 (4.0; 10.8)	6.5 (4.0; 9.0)	.862
≥1 fall during the observation period at T0	16	12	.300
MMSE	26.1 (24.0; 27.7)	26.4 (25.3; 27.2)	.000
Total LEDD	667.1 (500; 780)	700.0 (356.3; 900.0)	.551
L-dopa LEDD	487.5 (315.0; 690.0)	450.0 (293.8; 600.0)	.892
DA LEDD	120.0 (0.0; 275.0)	175.0 (0.0; 300.0)	.721

Abbreviations: * Mann-Witney U test was used for the comparisons except gender, which was compared with chi-square; H&Y: Hoehn & Yahr stage; LEDD: levodopa equivalent daily dose; MMSE: mini-mental state examination.

**Table 2:** Static stabilometry values of the two groups enrolled in the study at each time point.

		Equistasi			Control			T1-T0		T2-T0		T2-T1	
		T0	T1	T2	T0	T1	T2	Equistasi	Control	Equistasi	Control	Equistasi	Control
**Sway** **area (mm^2^)**	EO	**130.6** **(97.6; 400.2)**	192.0(95.2; 486.4)	**298.9** (175.2; 473.6)*	181.3(84.6; 351.8)	165.7(73.6; 743.4)	149.1(62.9; 331.6)	25.2(−141.2; 99.6)	−15.7(−126.5; 377.1)	**155.0** **(88.2; 244.0)**	−**74.3** **(**−**139.7;** 115.8)**	105.2(−21.3; 299.4)	−38.9(−345.2; 50.8)
	EC	337.5(90.3 803.8)	257.9(116.3; 892.5)	275.9(142.5; 565.7)	163.2(73.3; 664.1)	246.9(60.7; 595.9)	217.1(105.7; 384.0)	6.0(−314.4; 189.2)	15.8(−157.5; 212.9)	41.6(−289.4; 191.5)	31.6(−96.2; 240.8)	73.8(−587.0; 172.9)	−8.8(−207.2; 160.8)
**Sway** **path (mm)**	EO	418.2(278.9; 498.0)	353.5(274.2; 623.6)	409.3(309.0; 567.6)	347.1(295.0; 543.1)	351.9(275.2; 516.7)	314.2(224.5; 606.8)	−22.4(−113.6; 160.7)	2.2(−111.2; 79.5)	51.3(−92.0; 136.7)	−35.7(−153.5; 103.6)	31.0(−66.9; 82.4)	5.8(−68.6; 78.8)
	EC	571.8(321.6; 717.2)	530.3(357.0; 850.2)	461.1(304.3; 774.7)	440.0(308.4; 704.6)	453.0(294.7; 744.9)	414.0(290.2; 883.6)	−13.7(−146.6; 80.5)	−8.2(−110.9; 138.0)	30.1(−195.3; 91.2)	49.5(−119.1; 121.4)	35.1(−110.3; 59.4)	22.8(−46.8; 93.2)
**Mean COP** **Velocity (m/s)**	EO	8.2(5.4; 9.7)	6.9(5.4; 12.2)	8.0(6.0; 11.1)	6.8(5.8; 10.6)	6.9(5.4; 10.1)	6.1(4.4; 11.9)	−0.4(−2.2; 3.1)	0.0(−2.2; 1.5)	1.0(−1.8; 2.7)	−0.7(−3.0; 2.1)	0.6(−1.3; 1.6)	0.1(−1.3; 1.6)
	EC	11.2(6.3; 14.0)	10.3(7.0; 16.6)	9.0(5.9; 15.1)	8.6(6.0; 13.7)	8.8(5.7; 14.5)	8.1(5.7; 17.2)	−0.2(−2.9; 1.6)	−0.2(−2.2; 2.7)	0.6(−3.8; 1.8)	1.0(−1.7; 2.4)	0.7(−2.2; 1.2)	0.9(−0.9; 1.8)

Abbreviations: *: significantly different than T0 (p = .008); **: significantly different than Equistasi (p = .011); COP: centre of pressure; EC: eyes closed; EO: eyes open.

### Therapy outcomes in the Equistasi group

The combined intervention of balance training plus Equistasi did not modify the parameters of static posturography with the exception of the sway area during EO condition (p = 0.005); on post-hoc T2 these values were significantly lower than T0 (p = 0.008; Table 2). By contrast, a profound effect was found for both the sway area (p = 0.049) and the displacement along the AP axis (p = 0.039) at the instrumental FRT during the EC condition; on post-hoc, these were significantly higher at T1 than T0 (Figure 3).

As for the other clinical measures, a significant effect was found for UPDRS-II (p<0.001), UPDRS-III (p<0.001), BBS (p<0.001), TUG (p<0.001), ABC (p<0.001), FES (p<0.001), and PDQ-39 (p<0.001). Table 3 details the post-hoc results: overall, a significant improvement was observed at T1 for all the aforementioned scales; at T2 the improvement was retained for the UPDRS-III, BBS, TUG and PDQ-39 whereas UPDRS-II, ABC and FES were comparable than T0 and significantly worse than T1. Finally, a significant effect was found for the falls rate (p<0.001); on post-hoc analysis a significant improvement was found when comparing T1 and T2 with T0 (Figure 4A).

**Table 3:** Clinical variables of the two groups enrolled in the study and their comparisons at each time point.

	Equistasi						Control						T1 - T0			T2 - T0			T2 - T1		
	T0	T1	T2	T1 vs.T0	T2 vs.T0	T2 vs.T1	T0	T1	T2	T1 vs.T0	T2 vs.T0	T2 vs.T1	Equistasi	Control	p	Equistasi	Control	p	Equistasi	Control	p
UPDRS-II*	20.5(15.5; 25.3)	15.5(12.0; 19.8)	17.5(15.0; 22.8)	**.001**	.058	**.001**	18.5(15.3; 22.8)	14.5(10.0; 18.0)	18.0(12.0; 20.0)	**.001**	**.004**	**.001**	−3.0(−7.0; −1.3)	−4.0(−6.8; −4.0)	.050	−2.0(−4.0; 1.0)	−2.0(−3.0; −1.0)	.659	2.5(1.3; 4.8)	3.0(1.0; 4.0)	.730
UPDRS-III*	42.0(36.3; 48.3)	36.0(24.0; 39.8)	37.0(28.0; 43.8)	**.001**	**.015**	**.001**	39.5(31.3; 51.0)	33.5(23.5; 40.5)	38.0(32.0; 43.0)	**.001**	.071	**.001**	−7.0(−11.8; −3.3)	−8.0(−11.0; −4.3)	1.0	−2.0(−7.3; 0.0)	−3.0(−5.0; 1.0)	.756	6.0(2.0; 8.0)	4.0(1.0; 9.0)	.621
BBS∧	43.5(35.3; 45.0)	52.0(48.0; 54.0)	47.0(39.0; 49.0)	**.001**	**.002**	**.001**	45.5(40.0; 48.0)	51.0(47.5; 53.8)	49.0(45. 0; 51.0)	**.001**	**.001**	**.001**	**10.0** **(6.0; 13.0)**	**5.5** **(4.0; 7.8)**	**.026**	4.0(1.0; 6.0)	3.0(1.0; 4.0)	.745	−5.0(−9.0; −1.0)	−2.0(−4.0; −2.0)	.052
TUG*	14.6(12.2; 19.1)	12.4(10.7; 15.0)	12.7(12.2; 15.6)	**.001**	**.004**	**.005**	12.8(10.5; 14.7)	11.9(10.2; 13.3)	12.5(10.9; 13.9)	**.001**	**.022**	**.021**	−1.4(−6.3; −0.2)	−0.8(−1.4; −0.4)	.752	−0.8(−4.8; −0.2)	−0.6(−1.0; 0.1)	.516	0.7(0.1; 1.6)	0.4(0.1; 1.0)	.330
ABC∧	51.6(42.6; 61.5)	66.3(57.2; 82.2)	51.3(41.3; 73.1)	**.001**	.398	**.001**	56.2(52.5; 64.7)	59.1(53.8; 67.5)	51.3(41.9; 62.5)	**.012**	.444	**.002**	**15.3** **(5.9; 22.7)**	**2.5** **(**−**0.5; 7.3)**	**.004**	1.4(−8.1; 13.6)	−1.9(−11.3; 8.1)	.516	−12.8(−18.4; −8.1)	−3.3(14.4; −0.9)	.194
FES*	12.5(9. 5; 17.5)	6.5(3.3; 11.8)	12.0(6.0; 14.0)	**.001**	.063	**.006**	10.0(6.3; 15.0)	7.0(5.0; 12.0)	10.0(4.0; 14.0)	**.001**	.358	.405	−**6.0** **(**−**8.8; −2.5)**	−**2.0** **(**−**3.0; −1.0)**	**.026**	−2.0(−6.0; 1.0)	0.0(−5.0; 2.0)	.745	3.0(1.0; 7.0)	2.0(−2.0; 3.0)	.194
PDQ-39*	66.0(48.8; 73.3)	39.0(32.5; 60.5)	53.0(40.0; 68.0)	**.001**	**.039**	**.007**	63.0(36.3; 85.0)	58.0(35.0; 82.5)	59.0(40.0; 84.0)	**.001**	**0.039**	**.001**	−**13.5** **(**−**30. 5; −9.3)**	−**7.0** **(**−**9.8; −3.5)**	**.004**	−6.0(−20.0; 0.0)	−3.0(−6.0; 0.0)	.737	11.0(6.0; 20.0)	3.0(1.0; 7.0)	.052

Bold-typed values represent significant improvement; abbreviations: *: score reduction means improvement; ∧: score increase means improvement; ABC: Activities-specific Balance Confidence; BBS: Berg balance scale; FES: Falls Efficacy Scale; TUG: timed up and go, UPDRS: unified PD rating scale.

### Therapy outcomes in the Control group

The combined intervention of balance training plus placebo devices did not modify the parameters of static posturography nor of the instrumental FRT (Table 2 and Figure 3).

As for the other clinical measures, a significant effect was found for UPDRS-II (p<0.001), UPDRS-III (p<0.001), BBS (p<0.001), TUG (p<0.001), ABC (p = 0.006), FES (p = 0.004), and PDQ-39 (p<0.001). Table 3 details the post-hoc results: overall, a significant improvement was observed at T1 for all the aforementioned scales; at T2 the improvement was retained for the UPDRS-II and BBS, whereas FES, UPDRS-III and ABC were comparable to T0, being the last two also significantly worse than T1; TUG and PDQ-39 at T2 were significantly worse than T1 and T0 (Table 3).

No significant effect was found for the falls rate.

### Comparisons between Equistasi and Control groups

At T1, Equistasi group significantly presented less falls (Figure 4A). The same effect was detected when considering only fallers at T0 (p<.0001): in the Equistasi group median falls rate dropped from 4 (3; 4) at T0 to 0 (0; 1) at T1 and worsened again at T2 [4 (1; 3.75)]; by contrast, in the Control group median falls rate did not change, being 3 (2; 5.5), 3 (2; 4.5) and 3 (2; 5) at T0, T1 and T2, respectively (Figure 4B).

When comparing the between-group magnitude of change, Equistasi patients showed a significant improvement of at T1 in terms of BBS (+45.0%, p = .026), ABC (+83.7, p = .004), FES (−33.3%, p = .026) and PDQ-39 (−48.8%, p = .004) (Table 3). No other significant differences were found.

## Discussion

The present RCT has shown that enhancing balance training using a WPS in a rehabilitation setting is safe and might lead to some clinically meaningful effect in PD patients with balance impairment. Although both groups with an active and placebo WPS improved at the end of the rehabilitation period and we did not find significant between-group differences in the principal posturographic measures with the exception of higher sway area and limit of stability on the instrumental functional reach test during visual deprivation at T1, we found an overall improvement in many secondary endpoints only in patients enrolled in the Equistasi group. Specifically, we found a significant improvement at T1 on BBS, ABC, FES and PDQ-39, a sustained improvement at T2 of UPDRS-III, BBS, TUG and PDQ-39, and a significant and sustained reduction of the falls rate. By contrast, patients enrolled in the control group did not experience any falls rate reduction and at T2 only retained the improvement of UPDRS-II and BBS, whereas TUG and PDQ-39 were also significantly worse than baseline.

The rehabilitative programs were delivered successfully with high adherence in both groups and, in keeping with the current knowledge [27], balance training significantly improved the key clinical variables in both groups.

Posturography measurements were the principal endpoint of this RCT, given the higher sensitivity and reliability of instrumental results in trials with small sample size. The lack of between-groups differences at the baseline confirms the goodness of randomization already seen on clinical scales. Noteworthy, at the end of rehabilitation statistically significant differences arose in the Equistasi group only. The significant reduction at T2 of the sway area in static-EO condition is difficult to comment in absence of a group of healthy controls and, more importantly, in light of the lack of conclusive data linking static posturography with balance performance and falls in PD patients [53]. In fact, studies have shown either increased, normal or reduced spontaneous body sway [54], [55], indicating that reliability of postural sway during static conditions could be influenced by many factors such as the progression of the disease, pharmacological and non-pharmacological interventions, bradykinesia [56] or postural deformities [57]. Notwithstanding, the significant reduction of the sway area is in keeping with the reduction of the falls rate, since greater postural sway has shown to be a predictor of falls in PD patients [58].

PD patients have a reduced limit of stability particularly during dynamic conditions, thus pointing to dynamic posturography as a better instrument to capture improvement of balance [7], [8]. Accordingly, our RCT did show a significant increase of the limits of stability in the AP plane at T1 and only in the Equistasi group. Interestingly, the effect was only noticeable in the EC condition, a setting relying on the integrity of the vestibular and proprioceptive system, in keeping with the notion that bilateral Achilles tendon vibration applied in healthy subjects results in a profound effect on trunk, hips and knees displacement in the absence of vision [41]. The limit of stability mainly depends on the size of anticipatory postural adjustments, which have been found to be increased by Achilles tendon vibration in rectus femoris, biceps femoris and erector spinae muscles prior to a fast arm movement [59].

Spindles respond to vibrations as if the muscle is stretched, thus producing a tonic contraction on the stimulated muscle [60], [61]. Muscle vibration does not only impact on local spinal cord circuits but it also provides a substantial proprioceptive inflow to different parts of the central nervous system, thus influencing the accuracy of calibrations of action selection and execution of voluntary movement [62]. Accordingly, vibration of axial muscles has been proved to produce systematic changes in standing posture [16] and body orientation (the so-called “vibratory myesthetic illusions”) [63].

From the aforementioned studies, eliciting proprioceptive inflow by means of vibration has a biological plausibility in patients with PD, although the mechanism for its beneficial effect is largely speculative. Our findings are in keeping with previous researches using vibratory stimulation on trunk muscles for therapeutic purposes in PD, providing an improvement of trunk sway [35] or gait [21]. These studies have proved that patterned muscle vibrations are able to improve weight transfer along AP or ML axes, whose impairment is a core feature of axial control of PD patients [64]. In order to deliver vibration trains to the muscles, available studies have adopted battery-operated custom-made systems (generally consisting in vibrating units fixed on the distal tendons by elastic bands and connected to a wearable control unit) [21], [35]; by contrast, the WPS used in our protocol can be worn for days given the small dimension and the lack of power supply. Therefore, our experimental set up shows for the first time the effects of prolonged chronic externally applied vibrations. On the other hand, PD patients with postural instability respond hyperactively to proprioceptive sensory manipulation when a mechanical vibration is applied to the soleus muscles [24], thus raising the possibility that the prolonged use of vibration could have impaired balance. In addition, vibration has been shown to change spatial body orientation very fast [41], thus resulting in a postural response known as a “vibration-induced falling” [15], especially when vibration is used to experimentally impair proprioception. In contrast with such assumptions, we found a significant reduction of the falls rate, thus confirming that the positive effects found on the instrumental FRT are clinically meaningful and that the high-frequency and small-amplitude vibration induced by the WPS used in our protocol does not exert detrimental effects on proprioception. PD patients display an impairment of scaling and habituation of postural reactions triggered by lower leg proprioception, whereas they do not seem to present deficits in proprioceptive afferent or central integrative functions [24]. Notwithstanding, PD-related abnormalities in proprioception might manifest as alteration of kinesthesia, defined as the conscious awareness of body and limb position, motion and orientation in space (for a review see [65]). In addition, PD patients have an impaired sense of the timing [66] and discrimination [67] of proprioceptive inputs, which can also lead to deficient compensation of mechanical perturbations. The enhancement of the proprioceptive inflow, as that induced by the present vibration protocol with Equistasi, might overcome the subtle impairment in kinesthesia, as previously argued [20]. Another potential mechanism of action could be related to the influence on muscular tone, since tendon vibration has been successfully applied in healthy subjects to change muscular tone during voluntary contraction [68] or to improve muscle velocity and strength after training [69].

The improvement of falls rate was retained 2 months after rehabilitation, thus supporting a strong and synergic effect between Equistasi and balance training. Falling is a major determinant of quality and quantity of life in PD patients, but it is often resistant to classic antiparkinsonian treatment and different approaches are currently tried, ranging from medications enhancing the central cholinergic pathways to DBS targeting brainstem nuclei (for a review see [5]). Since rehabilitation still remains the main treatment for balance problems, but it is often ineffective in the long-term, the sustained benefit on the falls rate detected by our experimental protocol deserves attention and needs to be replicated by future studies.

This pilot study has a number of limitations. First, even if testing occurred at peak dose of the morning medications, we cannot rule out the bias introduced by fluctuations in levodopa plasmatic concentration; however, this limitation plays a marginal role in the study results because: 1) it applies to both groups, 2) it is well known that levodopa does not hugely impact on posturography, 2) we excluded patients with dyskinesias, and 4) instrumental changes were paralleled by changes in other clinical measures relying on historical data. Second, though the sample size allowed the detection of significant changes, it is small and our results have to be replicated by larger trial, possibly enrolling patients with an higher number of falls at baseline. Third, even if the physiotherapy program for balance training was conducted in according with published guidelines, the execution of exercises were influenced by therapists expertise and patients’ motivation, meaning that our protocol does not necessarily reflect the clinical practice in other parts of the world. Finally, WPS were only tested on the neck and soleus muscles and not in other muscles involved in posture control; in fact, it had been demonstrated that vibration applied to the ankle or trunk muscles alone produces different effects on posture or gait [70]; in addition, it is known that these effects are modulated by the frequency of stimulation (typically 100–200 Hz) and to the best of our knowledge no study has employed the very high frequency delivered by Equistasi. Future protocols should compare the effects of vibrations applied on different muscles as well as different frequencies of vibration.

In conclusion, this pilot RCT shows that a physiotherapy program based on perturbation-based training in association with focal mechanical vibration exerted by a wearable postural stabilizer appears to be safe and tolerated; in addition, although it fails to prove superiority in most of primary endpoints, it resulted effective in improving clinical variables assessing self-confidence of balance, disability and falls rate, overall positively impacting on the health related quality of life. This preliminary investigation provides encouraging data on the feasibility and safety of our protocol, thus supporting the development of a large scale RCT. Future studies are certainly needed and will expand our knowledge on the mechanisms of action of WPS, on the exposure time needed to achieve a meaningful improvement and on its long-term duration.

## Supporting Information

Table S1 **Table S1 details the type of daily physiotherapy provided by the hospital physiotherapists.**
(DOC)

Protocol S1 **The study protocol.**
(DOCX)

Checklist S1 **CONSORT checklist.**
(DOCX)

## References

[1] Lamont RM, Morris ME, Woollacott MH, Brauer SG (2012) Community walking in people with Parkinson’s disease. Parkinsons Dis 2012: 856237. 22191078 10.1155/2012/856237 PMC3236447

[2] Tan D, Danoudis M, McGinley J, Morris ME (2012) Relationships between motor aspects of gait impairments and activity limitations in people with Parkinson’s disease: a systematic review. Parkinsonism Relat Disord 18: 117–124. 22093237 10.1016/j.parkreldis.2011.07.014

[3] Soh SE, Morris ME, McGinley JL (2011) Determinants of health-related quality of life in Parkinson’s disease: a systematic review. Parkinsonism Relat Disord 17: 1–9. 20833572 10.1016/j.parkreldis.2010.08.012

[4] Benatru I, Vaugoyeau M, Azulay JP (2008) Postural disorders in Parkinson’s disease. Neurophysiol Clin 38: 459–465. 19026965 10.1016/j.neucli.2008.07.006

[5] Fasano A, Plotnik M, Bove F, Berardelli A (2012) The neurobiology of falls. Neurol Sci 33: 1215–1223. 22673818 10.1007/s10072-012-1126-6

[6] Menant JC, Latt MD, Menz HB, Fung VS, Lord SR (2011) Postural sway approaches center of mass stability limits in Parkinson’s disease. Mov Disord 26: 637–643. 21312283 10.1002/mds.23547

[7] Schieppati M, Hugon M, Grasso M, Nardone A, Galante M (1994) The limits of equilibrium in young and elderly normal subjects and in parkinsonians. Electroencephalogr Clin Neurophysiol 93: 286–298. 7521289 10.1016/0168-5597(94)90031-0

[8] Nonnekes J, de Kam D, Geurts A, Weerdesteyn V, Bloem BR (2013) Unraveling the mechanisms underlying postural instability in Parkinson’s disease using dynamic posturography. Expert Rev Neurother 13: 1303–1308. 24160682 10.1586/14737175.2013.839231

[9] Beckley DJ, Bloem BR, Remler MP (1993) Impaired scaling of long latency postural reflexes in patients with Parkinson’s disease. Electroencephalogr Clin Neurophysiol 89: 22–28. 7679626 10.1016/0168-5597(93)90080-9

[10] Peterka RJ (2002) Sensorimotor integration in human postural control. J Neurophysiol 88: 1097–1118. 12205132 10.1152/jn.2002.88.3.1097

[11] Mohapatra S, Krishnan V, Aruin AS (2012) Postural control in response to an external perturbation: effect of altered proprioceptive information. Exp Brain Res 217: 197–208. 22198575 10.1007/s00221-011-2986-3 PMC3325787

[12] Schieppati M, Crenna P (1984) From activity to rest: gating of excitatory autogenetic afferences from the relaxing muscle in man. Exp Brain Res 56: 448–457. 6499972 10.1007/BF00237985

[13] Desmedt JE, Godaux E (1978) Mechanism of the vibration paradox: excitatory and inhibitory effects of tendon vibration on single soleus muscle motor units in man. J Physiol 285: 197–207. 154563 10.1113/jphysiol.1978.sp012567 PMC1281752

[14] Burke D, Hagbarth KE, Lofstedt L, Wallin BG (1976) The responses of human muscle spindle endings to vibration during isometric contraction. J Physiol 261: 695–711. 135841 10.1113/jphysiol.1976.sp011581 PMC1309167

[15] Eklund G (1972) General features of vibration-induced effects on balance. Ups J Med Sci 77: 112–124. 4262735 10.1517/03009734000000016

[16] Courtine G, De Nunzio AM, Schmid M, Beretta MV, Schieppati M (2007) Stance- and locomotion-dependent processing of vibration-induced proprioceptive inflow from multiple muscles in humans. J Neurophysiol 97: 772–779. 17065250 10.1152/jn.00764.2006

[17] Smiley-Oyen AL, Cheng HY, Latt LD, Redfern MS (2002) Adaptation of vibration-induced postural sway in individuals with Parkinson’s disease. Gait Posture 16: 188–197. 12297259 10.1016/s0966-6362(02)00005-x

[18] Nardone A, Schieppati M (2005) Reflex contribution of spindle group Ia and II afferent input to leg muscle spasticity as revealed by tendon vibration in hemiparesis. Clin Neurophysiol 116: 1370–1381. 15978499 10.1016/j.clinph.2005.01.015

[19] Bove M, Nardone A, Schieppati M (2003) Effects of leg muscle tendon vibration on group Ia and group II reflex responses to stance perturbation in humans. J Physiol 550: 617–630. 12777449 10.1113/jphysiol.2003.043331 PMC2343054

[20] De Nunzio AM, Nardone A, Picco D, Nilsson J, Schieppati M (2008) Alternate trains of postural muscle vibration promote cyclic body displacement in standing parkinsonian patients. Mov Disord 23: 2186–2193. 18785234 10.1002/mds.22201

[21] De Nunzio AM, Grasso M, Nardone A, Godi M, Schieppati M (2010) Alternate rhythmic vibratory stimulation of trunk muscles affects walking cadence and velocity in Parkinson’s disease. Clin Neurophysiol 121: 240–247. 19955020 10.1016/j.clinph.2009.10.018

[22] Demirci M, Grill S, McShane L, Hallett M (1997) A mismatch between kinesthetic and visual perception in Parkinson’s disease. Ann Neurol 41: 781–788. 9189039 10.1002/ana.410410614

[23] Valkovic P, Krafczyk S, Saling M, Benetin J, Botzel K (2006) Postural reactions to neck vibration in Parkinson’s disease. Mov Disord 21: 59–65. 16149087 10.1002/mds.20679

[24] Valkovic P, Krafczyk S, Botzel K (2006) Postural reactions to soleus muscle vibration in Parkinson’s disease: scaling deteriorates as disease progresses. Neurosci Lett 401: 92–96. 16574321 10.1016/j.neulet.2006.02.073

[25] Li F, Harmer P, Fitzgerald K, Eckstrom E, Stock R, et al (2012) Tai chi and postural stability in patients with Parkinson’s disease. N Engl J Med 366: 511–519. 22316445 10.1056/NEJMoa1107911 PMC3285459

[26] Hirsch MA, Toole T, Maitland CG, Rider RA (2003) The effects of balance training and high-intensity resistance training on persons with idiopathic Parkinson’s disease. Arch Phys Med Rehabil 84: 1109–1117. 12917847 10.1016/s0003-9993(03)00046-7

[27] Allen NE, Sherrington C, Paul SS, Canning CG (2011) Balance and falls in Parkinson’s disease: a meta-analysis of the effect of exercise and motor training. Mov Disord 26: 1605–1615. 21674624 10.1002/mds.23790

[28] Ashburn A, Fazakarley L, Ballinger C, Pickering R, McLellan LD, et al (2007) A randomised controlled trial of a home based exercise programme to reduce the risk of falling among people with Parkinson’s disease. J Neurol Neurosurg Psychiatry 78: 678–684. 17119004 10.1136/jnnp.2006.099333 PMC2117667

[29] Goodwin VA, Richards SH, Henley W, Ewings P, Taylor AH, et al (2011) An exercise intervention to prevent falls in people with Parkinson’s disease: a pragmatic randomised controlled trial. J Neurol Neurosurg Psychiatry 82: 1232–1238. 21856692 10.1136/jnnp-2011-300919

[30] Morris ME, Iansek R, Kirkwood B (2009) A randomized controlled trial of movement strategies compared with exercise for people with Parkinson’s disease. Mov Disord 24: 64–71. 18942100 10.1002/mds.22295

[31] Munneke M, Nijkrake MJ, Keus SH, Kwakkel G, Berendse HW, et al (2010) Efficacy of community-based physiotherapy networks for patients with Parkinson’s disease: a cluster-randomised trial. Lancet Neurol 9: 46–54. 19959398 10.1016/S1474-4422(09)70327-8

[32] Rochester L, Rafferty D, Dotchin C, Msuya O, Minde V, et al (2010) The effect of cueing therapy on single and dual-task gait in a drug naive population of people with Parkinson’s disease in northern Tanzania. Mov Disord 25: 906–911. 20175212 10.1002/mds.22978

[33] Morris ME (2006) Locomotor training in people with Parkinson disease. Phys Ther 86: 1426–1435. 17012646 10.2522/ptj.20050277

[34] Morris ME, Martin CL, Schenkman ML (2010) Striding out with Parkinson disease: evidence-based physical therapy for gait disorders. Phys Ther 90: 280–288. 20022998 10.2522/ptj.20090091 PMC2816030

[35] Nanhoe-Mahabier W, Allum JH, Pasman EP, Overeem S, Bloem BR (2012) The effects of vibrotactile biofeedback training on trunk sway in Parkinson’s disease patients. Parkinsonism Relat Disord 18: 1017–1021. 22721975 10.1016/j.parkreldis.2012.05.018

[36] Equistasi website. Available: http://www.equistasi.com/en/. Accessed 2013 Dec 13.

[37] Necking LE, Lundstrom R, Dahlin LB, Lundborg G, Thornell LE, et al (1996) Tissue displacement is a causative factor in vibration-induced muscle injury. J Hand Surg Br 21: 753–757. 8982917 10.1016/s0266-7681(96)80180-x

[38] Folstein MF, Folstein SE, McHugh PR (1975) “Mini-mental state”. A practical method for grading the cognitive state of patients for the clinician. J Psychiatr Res 12: 189–198. 1202204 10.1016/0022-3956(75)90026-6

[39] Berardelli A, Wenning GK, Antonini A, Berg D, Bloem BR, et al (2013) EFNS/MDS-ES/ENS [corrected] recommendations for the diagnosis of Parkinson’s disease. Eur J Neurol 20: 16–34. 23279440 10.1111/ene.12022

[40] Hoehn MM, Yahr MD (1967) Parkinsonism: onset, progression and mortality. Neurology 17: 427–442. 6067254 10.1212/wnl.17.5.427

[41] Thompson M, Medley A (2007) Forward and lateral sitting functional reach in younger, middle-aged, and older adults. J Geriatr Phys Ther 30: 43–48. 18171486 10.1519/00139143-200708000-00002

[42] Nieuwboer A, Rochester L, Muncks L, Swinnen SP (2009) Motor learning in Parkinson’s disease: limitations and potential for rehabilitation. Parkinsonism Relat Disord 15 Suppl 3: S53–58. 10.1016/S1353-8020(09)70781-3 20083008

[43] Scoppa F, Capra R, Gallamini M, Shiffer R (2013) Clinical stabilometry standardization: basic definitions-acquisition interval–sampling frequency. Gait Posture 37: 290–292. 22889928 10.1016/j.gaitpost.2012.07.009

[44] Duncan PW, Weiner DK, Chandler J, Studenski S (1990) Functional reach: a new clinical measure of balance. J Gerontol 45: M192–197. 2229941 10.1093/geronj/45.6.m192

[45] Abdo WF, Borm GF, Munneke M, Verbeek MM, Esselink RA, et al (2006) Ten steps to identify atypical parkinsonism. J Neurol Neurosurg Psychiatry 77: 1367–1369. 16847047 10.1136/jnnp.2006.091322 PMC2077404

[46] Fahn S, Elton R, Members of the UPDRS Development Committee (1987) Recent developments in Parkinson’s disease. Fahn S, Marsden C, Calne D, Goldstein M, editors. Folorham Park, NJ: Macmillan Health Care Information. 153–163, 293–304 p.

[47] Podsiadlo D, Richardson S (1991) The timed “Up & Go”: a test of basic functional mobility for frail elderly persons. J Am Geriatr Soc 39: 142–148. 1991946 10.1111/j.1532-5415.1991.tb01616.x

[48] Berg K, Wood-Dauphinee S, Williams JI (1995) The Balance Scale: reliability assessment with elderly residents and patients with an acute stroke. Scand J Rehabil Med 27: 27–36. 7792547

[49] Tinetti ME, Richman D, Powell L (1990) Falls efficacy as a measure of fear of falling. J Gerontol 45: P239–243. 2229948 10.1093/geronj/45.6.p239

[50] Powell LE, Myers AM (1995) The Activities-specific Balance Confidence (ABC) Scale. J Gerontol A Biol Sci Med Sci 50A: M28–34. 7814786 10.1093/gerona/50a.1.m28

[51] Peto V, Jenkinson C, Fitzpatrick R, Greenhall R (1995) The development and validation of a short measure of functioning and well being for individuals with Parkinson’s disease. Qual Life Res 4: 241–248. 7613534 10.1007/BF02260863

[52] Tomlinson CL, Stowe R, Patel S, Rick C, Gray R, et al (2010) Systematic review of levodopa dose equivalency reporting in Parkinson’s disease. Mov Disord 25: 2649–2653. 21069833 10.1002/mds.23429

[53] Nardone A, Schieppati M (2006) Balance in Parkinson’s disease under static and dynamic conditions. Mov Disord 21: 1515–1520. 16817196 10.1002/mds.21015

[54] Schieppati M, Nardone A (1991) Free and supported stance in Parkinson’s disease. The effect of posture and ‘postural set’ on leg muscle responses to perturbation, and its relation to the severity of the disease. Brain 114 (Pt 3): 1227–1244. 2065247 10.1093/brain/114.3.1227

[55] Horak FB, Nutt JG, Nashner LM (1992) Postural inflexibility in parkinsonian subjects. J Neurol Sci 111: 46–58. 1402997 10.1016/0022-510x(92)90111-w

[56] Paul SS, Canning CG, Sherrington C, Fung VS (2012) Reproducibility of measures of leg muscle power, leg muscle strength, postural sway and mobility in people with Parkinson’s disease. Gait Posture 36: 639–642. 22640867 10.1016/j.gaitpost.2012.04.013

[57] Latt MD, Lord SR, Morris JG, Fung VS (2009) Clinical and physiological assessments for elucidating falls risk in Parkinson’s disease. Mov Disord 24: 1280–1289. 19425059 10.1002/mds.22561

[58] Kerr GK, Worringham CJ, Cole MH, Lacherez PF, Wood JM, et al (2010) Predictors of future falls in Parkinson disease. Neurology 75: 116–124. 20574039 10.1212/WNL.0b013e3181e7b688

[59] Slijper H, Latash ML (2004) The effects of muscle vibration on anticipatory postural adjustments. Brain Res 1015: 57–72. 15223367 10.1016/j.brainres.2004.04.054

[60] Roll JP, Vedel JP (1982) Kinaesthetic role of muscle afferents in man, studied by tendon vibration and microneurography. Exp Brain Res 47: 177–190. 6214420 10.1007/BF00239377

[61] Marsden CD, Meadows JC, Hodgson HJ (1969) Observations on the reflex response to muscle vibration in man and its voluntary control. Brain 92: 829–846. 4312217 10.1093/brain/92.4.829

[62] Kording KP, Wolpert DM (2006) Bayesian decision theory in sensorimotor control. Trends Cogn Sci 10: 319–326. 16807063 10.1016/j.tics.2006.05.003

[63] Lackner JR, Levine MS (1979) Changes in apparent body orientation and sensory localization induced by vibration of postural muscles: vibratory myesthetic illusions. Aviat Space Environ Med 50: 346–354. 464954

[64] Rocchi L, Chiari L, Mancini M, Carlson-Kuhta P, Gross A, et al (2006) Step initiation in Parkinson’s disease: influence of initial stance conditions. Neurosci Lett 406: 128–132. 16901637 10.1016/j.neulet.2006.07.027

[65] Conte A, Khan N, Defazio G, Rothwell JC, Berardelli A (2013) Pathophysiology of somatosensory abnormalities in Parkinson disease. Nat Rev Neurol 9: 687–697. 24217516 10.1038/nrneurol.2013.224

[66] Fiorio M, Stanzani C, Rothwell JC, Bhatia KP, Moretto G, et al (2007) Defective temporal discrimination of passive movements in Parkinson’s disease. Neurosci Lett 417: 312–315. 17367930 10.1016/j.neulet.2007.02.050

[67] Jacobs JV, Horak FB (2006) Abnormal proprioceptive-motor integration contributes to hypometric postural responses of subjects with Parkinson’s disease. Neuroscience 141: 999–1009. 16713110 10.1016/j.neuroscience.2006.04.014

[68] Cordo P, Gurfinkel VS, Bevan L, Kerr GK (1995) Proprioceptive consequences of tendon vibration during movement. J Neurophysiol 74: 1675–1688. 8989404 10.1152/jn.1995.74.4.1675

[69] Bosco C, Colli R, Introini E, Cardinale M, Tsarpela O, et al (1999) Adaptive responses of human skeletal muscle to vibration exposure. Clin Physiol 19: 183–187. 10200901 10.1046/j.1365-2281.1999.00155.x

[70] Ivanenko YP, Grasso R, Lacquaniti F (2000) Influence of leg muscle vibration on human walking. J Neurophysiol 84: 1737–1747. 11024066 10.1152/jn.2000.84.4.1737

