# Supplementary material for: A Wearable Proprioceptive Stabilizer (Equistasi®) for Rehabilitation of Postural Instability in Parkinson’s Disease: A Phase II Randomized Double-Blind, Double-Dummy, Controlled Study
Source: PLoS One. 2014 Nov 17;9(11):e112065. doi: 10.1371/journal.pone.0112065 (PMC4234681; doi:10.1371/journal.pone.0112065)
Supplement: Table S1 — details the type of daily physiotherapy provided by the hospital physiotherapists. (DOC) [file pone.0112065.s001.doc]

**Table S1.** The physiotherapy program for balance training in according to the KNGF guidelines for physical therapy in Parkinson’s disease (<http://www.appde.eu/european-physiotherapy-guidelines.asp>).

| **Domain** | **Goal** |
| --- | --- |
| **Improvement of physical capacity:** | To maintain or to improve physical capacity with training of aerobic muscle strength (with the emphasis on the muscles of the trunk and legs), joint mobility (among others, axial) and muscle length (among others, muscles of the calf and hamstrings, flexor and extensor of the knee) |
| **Improvement of the transfers:** | To train transfers by applying cognitive movement strategies and cues to initiate and continue movement |
| **Normalizing body posture:** | To preventing or treating postural deformities with exercises for postural realignment and coordinated movements |
| **Training balance** | To optimize balance during the performance of activities in static and dynamic conditions with exercises for training strength and pertubation-based balance training with emphasis on functional reaching test in protected condition and how to activate postural responses to pertubation. Falls prevention strategies |
| **Gait training:** | To walk safely and to increase (comfortable) walking speed with exercise walking with the use of cues and cognitive movement strategies and to train muscle strength and mobility of the trunk and upper and lower limbs. |
