# Supplementary material for: A Wearable Proprioceptive Stabilizer (Equistasi®) for Rehabilitation of Postural Instability in Parkinson’s Disease: A Phase II Randomized Double-Blind, Double-Dummy, Controlled Study
Source: PLoS One. 2014 Nov 17;9(11):e112065. doi: 10.1371/journal.pone.0112065 (PMC4234681; doi:10.1371/journal.pone.0112065)
Supplement: Protocol S1 — The study protocol. (DOCX) [file pone.0112065.s002.docx]

**CLINICAL RESEARCH TRIAL**

**Venezia 04/06/2013**

**TITLE**

Rehabilitation of Postural Instability in Parkinson's disease: a randomized comparison between a new approach with an innovative proprioceptive device and physiotherapy

**A Wearable Proprioceptive Device (Equistasi^®^) as Postural Stabilizer for Rehabilitation of Postural Instability in Parkinson’s disease:** **A phase II Randomized Double-Blind, Double-Dummy, Controlled study**

Division in which the research is carried
U.O.S. Neurorehabilitation-San Raffaele Arcangelo Fatebenefratelli Venice

Responsible

Dr. Daniele Volpe MD e-mail [dott.dvolpe@libero.it](mailto:dott.dvolpe@libero.it)

Participating researchers:

Maria Giulia Giantin (PhD PT), Dr. Alfonso Fasano MD. PhD

**BACKGROUND**

Postural disorders in Parkinson's disease fit into the context of a general axial involvement , with a negative influence on balance and gait resulting in a significant worsening impact on quality of life . Some authors have described the various mechanisms involved in the development of these alterations and reported that there are multiple mechanisms that play an key role in the development of these postural disorders. In particular seems that there is a disintegration of proprioceptive information with a critical role of sensory information at different level of CSN with an impairment in kinesthesia. The aim of this pilot study is to verify whether a rehabilitation approach that involves the use of a device proprioceptive ( Equistasi ) is more effective than single traditional physiotherapy for postural deformities and postural instability. We believe that the use of this novel postural device(Equistasi) could balance and posture and that could help in maintaining long-term after the treatment . Equistasi is a Class I medical device CE mark issued by the Ministry of Health 30/10/2009 registered as a postural stabilizer . This is a device the size of 1 x 2 cm , composed exclusively of synthetic fibers coated with a shell of soft and flexible , non-toxic and CE marked , which is not powered by electric charges , no fibers deriving from metals , it is not chemical compound pharmacological , has no microchip inside. In contact with body temperature emits focal micromechanical vibrations that can not be perceived by the patient. Its action results in a stimulation of proprioception primarily via neuronal pathways that lead to the vibratory sensitivity (via spino- ponto - thalamic ) . Our hypothesis is supported by several studies demonstrating that in the neck there is a high concentration of proprioceptive receptors that are important for the maintenance of the head on the vertical line during upright standing position . It has also been demonstrated that patients may have a deficit in the perception of one's body position ,confirmed by the fact that the perception of horizontal and vertical position in patients with Parkinson's disease is characterized by a deviation in lateral bending. The study will use this postural stabilizer device ( Equistasi ) in association with a physiotherapy program for balance and posture because it seems to stimulate the proprioceptive information , thanks to the special composition of microfibers that emit micromechanical vibrations . The use of the latter has led, in recent studies , a number of positive effects in stimulating muscle proprioception and specifically on the use of focal mechanical vibratory stimulation in Parkinson's disease .

**OBJECTIVE**

The objective of this preliminary study is:

- to verify if the proposed device (Equistasi) as a proprioceptive postural stabilizer may represent an innovative and effective treatment in association with a physiotherapy program in improving balance and posture alterations and in maintenaning the outcome

-verify if the improvement obtained on postural perturbations have a positive effect on postural stability and falls in PD.

**STUDY DESIGN**

The study is expected to enroll approximately 36 patients; they will be randomized into one of the following groups :

1 . 18 patients Experimental Group E-PT ( Equistasi + Physical Therapy )

2 . 18 patients Group Control P -PT ( Placebo + Physical Therapy)

The two groups will submitt to the following programs :

Group 1 E-PT : physiotherapy program plus the innovative postural stabilizer device ( Equistasi ) for 8 weeks with a training balance program which includes exercises for balance training, postural realignment , muscle strengthening.

Equistasi will be applied on the skin with a duration ranging from a minimum of one hour to a maximum of four hours per day, gradually increasing the duration of application every week.

Group 2 P -PT : similar physiotherapy treatment plus an inactive device (placebo) , of the same duration and frequency of the first group . It will be used in the form of a placebo device similar to 'Equistasi but inactive.

**PARTICIPANTS ELIGIBILITY**

INCLUSION CRITERIA**:**

- Patients admitted to the U. O.S. Neurorehabilitation FBF Venice

- Parkinson's disease staged II-III H & Y

- Presence of postural alterations

- Presence of postural instability

- Ability to participate in a physiotherapy venue

- Absence of cognitive impairment (MMSE> 24/30)

- Stable medications

EXCLUSION CRITERIA:

- Presence of DBS

- severe cardiac and / or pulmonary disease

-Unstable medication

**STATISTICAL ANALYSIS**

They will be subjected to statistical analysis of the data derived from the stabilometric , posturographic evluation , as well as those from clinical scales . This clinical trial will use a sample of convenience, with the assumption that 40 participants would be ample to explore safety and feasibility. Given the small sample and the lack of normal distribution of most of the variables on Shapiro-Wilk test, non-parametric statistics will use. Initially, The data will be subjected to statistical analysis ANOVA in order to produce an analysis of variance univariate and then to test the hypothesis of equality of more medium .
A comparison will be made for all variables (extrapolated data from rating scales , posturographic test , and baropodometry stabilometry ) and for dependent samples for independent samples to evaluate both the variation within the group than those between the two groups. This will be done through the use of a T- test .
The number of patients included in the study is the minimum number to conduct a pilot study to have a statistical significance level . Patients will undergo randomization criteria following the Conshort Procedures for radomization.

**METHODS**

The following evaluation forms , questionnaires and instrumental evaluation will be administered at the beginning of treatment to all patients in all groups :

- Measurement of the angle of inclination of the trunk, using markers placed on specific points of reference on the basis of criteria established by analyzing posturographic

- Measurement of the position of the center of mass (COM) through data extracted from platform to baropodometry

- Stabilometry to evaluate the COP displacement in static and instrumental FRT

- Berg Balance Scale to assess balance

- Diary of falls and FES (Fall Efficacy Scale) to monitor falls rate and fear of falling

- PDQ -39 to assess the quality of life

- UPDRS (II -III section ) to assess motor disability

These instrumental tests , clinical evaluation will be administered again at the end of the eight weeks of treatment, rehabilitation and after another eight weeks of follow up.

Duration of the research

For each patient, an initial assessment will be performed T0, another evaluation after 8 weeks of treatment T1 and a final evaluation T2 after 8 weeks. The duration of the research study for each patient was then 16 weeks.

**Ethical considerations and evaluation of the benefit / risk ratio**

The benefit that patients will gain from participation in this study will be to receive a novel alternative and innovative treatment for postural alterations and balance problems , as it is proposed a typology of intervention based on the use of an innovative device Equistasi and furthermore a possible response to treatment of postural changes and balance problems , which currently represent a significant problem that can seriously affect the quality of life of patients with Parkinson's disease .
In addition, participating in the study, patients will make an important contribution to scientific research in the field of Parkinson's disease .
Patients included in the study will not change medications during the trial.
No risk has been described in the literature except for a skin allergy due to the patch which is used to apply the device.

**FUNDING**

Patients included in the study will not incur any costs as the device will be provided for free grant for scientific research by the Company Equistasi, also instrumental examinations and evaluations will be supported entirely by the Institute which will be held at the project research.
